# Phylogenetic analysis of the chloroplast genome for *Magnolia liliflora* (Magnoliaceae), an endemic species to China

**DOI:** 10.1080/23802359.2019.1664349

**Published:** 2019-09-12

**Authors:** Jiang Luo, Tian Ruan, Zhiping Wan

**Affiliations:** a Jiangxi Province Hospital of Integrated Chinese and Western Medicine, Nanchang, Jiangxi, China;; b Basic Medical School of Kunming Medical University, Second Clinical Medical School of Kunming Medical University, Kunming Medical University, Kunming, Yunnan, China;; c Nanchang Hongdu Hospital of Traditional Chinese Medicine, Nanchang, Jiangxi, China

**Keywords:** *Magnolia liliflora*, Magnoliaceae, endemic species, chloroplast genome, phylogenetic analysis

## Abstract

*Magnolia liliflora* is commonly called lily magnolia and its buds are used as the herbal medicine; it is an endemic species to China. In this study, the complete chloroplast genome of *M. liliflora* has been presented and annotated. The whole chloroplast (cp) genome is 158,177 bp in size, which exhibits a large single-copy region (LSC) of 88,134 bp, a small single-copy region (SSC) of 19,876 bp and two inverted-repeat regions (IRs) 25,706 bp in each one. The overall nucleotide composition is: 30.0% of A, 30.9% of T, 19.9% C, and 19.2% G, with the GC content of the chloroplast genome 39.1%. The cp genome of *M. liliflora* contains 129 genes, which includes 86 protein-coding genes (PCGs), 35 transfer RNA (tRNAs), and 8 ribosome RNA (rRNAs). The maximum-likelihood (ML) tree result showed that *M. liliflora* is closely related to two Magnoliaceae family species of *M. dealbata* and *M. glaucifolia* in phylogenetic relationship. This complete chloroplast genomes will be useful for medicinal study in the future.

*Magnolia liliflora* is the most important Magnoliaceae species that is commonly called lily magnolia in China, its buds are used in herbal medicine and it is an endemic species to China (Wang et al. 2012). It is native to the southwest China (in Sichuan and Yunnan), but cultivated for centuries elsewhere in China and also in Japan. *Magnolia liliflora* is a traditional flower and herbal medicine with a history of more than 2000 years in China, which has also been identified as vulnerable in the Red List of Chinese Species (Brach and Song 2006). Simultaneously, rare genomic data were reported about *M. liliflora*. In order to study the genetic diversity and genetic relationship of *M. liliflora* species, the cp genome of *M. liliflora* have been presented and studied, which will be useful for the medicinal study in the future.

The specimen sample of *M. liliflora* was collected from Jiangxi Province Hospital of Integrated Chinese and Western Medicine (Nanchang, Jiangxi, China, 115.91E; 28.67 N). Total genomic DNA of *M. liliflora* was extracted from the bud tissues using Plant Tissues Genomic DNA Extraction Kit (Solarbio, BJ and CN) and stored in the Jiangxi Province Hospital of Integrated Chinese and Western Medicine (No. JXPHICWM03). The chloroplast (cp) DNA was purified and fragmented using the NEB Next Ultra^TM^ II DNA Library Prep Kit (NEB, BJ and CN), which was later sequenced. Quality control was performed to remove low-quality reads and adapters using the FastQC software (Andrews 2015). The chloroplast genome was assembled and annotated using the MitoZ software (Meng et al. 2019). The physical map of the chloroplast genome was generated using OrganellarGenomeDRAW (Lohse et al. 2013). The accurate new annotated chloroplast genome had been submitted to the GenBank and the accession is No.MK9477631.

The complete chloroplast genome of *M. liliflora* is circular in shape with 158,177 base pairs (bp) in size, which harbors a characteristic quadripartite structure that has a large single-copy region (LSC) of 88,134 bp, a small single-copy region (SSC) of 18,743 bp, and two inverted repeat regions (IRs) of 25,650 bp. The cp genome of *M. liliflora* contains 129 genes, including 86 protein-coding genes (PCG), 35 transfer RNA genes (tRNAs), and 8 ribosomal RNA genes (rRNAs). The total of 18 genes were found duplicated in each one of the IR regions, which included 8 PCG species (*rpl2, rpl23, ycf2, ycf15, ndhB, rps7, rps12 a*nd *ycf1*), 6 tRNs species (*trnI-CAU, trnL-CAA, trnI-GAU, trnA-UGC, trnR-ACG* and *trnN-GUU*), and 4 rRNs species (*rRNA16, rRNA23, rRNA4.5* and *rRNA5*). The overall nucleotide composition is follows: 30.0% of A, 30.9% of T, 19.9% of C, and 19.2% of G, with the GC content of 39.1%.

Phylogenetic relationship analyses were performed based on two data partitions (complete cp DNA sequences and 39 protein-coding exons) from 20 plants cp genomes to construct the phylogenetic tree. The phylogenetic tree was reconstructed using the maximum-likelihood (ML) method. ML analysis was performed using the MEGA X software (Kumar et al. 2018) with 5000 bootstrap values replicate at each node based on the GTR model. All of the nodes were inferred with strong support by the ML methods. The final tree was represented using the MEGA X software (Kumar et al. 2018) and edited using the Evolview version 3.0 online web (www.evolgenius.info/evolview/) (Subramanian et al. 2019). Phylogenetic ML tree (Figure 1) result showed that the cp genome of *M. liliflora* is clustered and closest to two Magnoliaceae family species of *M. dealbata* and *M. glaucifolia* (GenBank No.NC 023235.1 and NC 037003.1) in the phylogenetic relationship. However, this study is very important and useful for medicinal valuable and clinical drug development.

**Figure 1.**
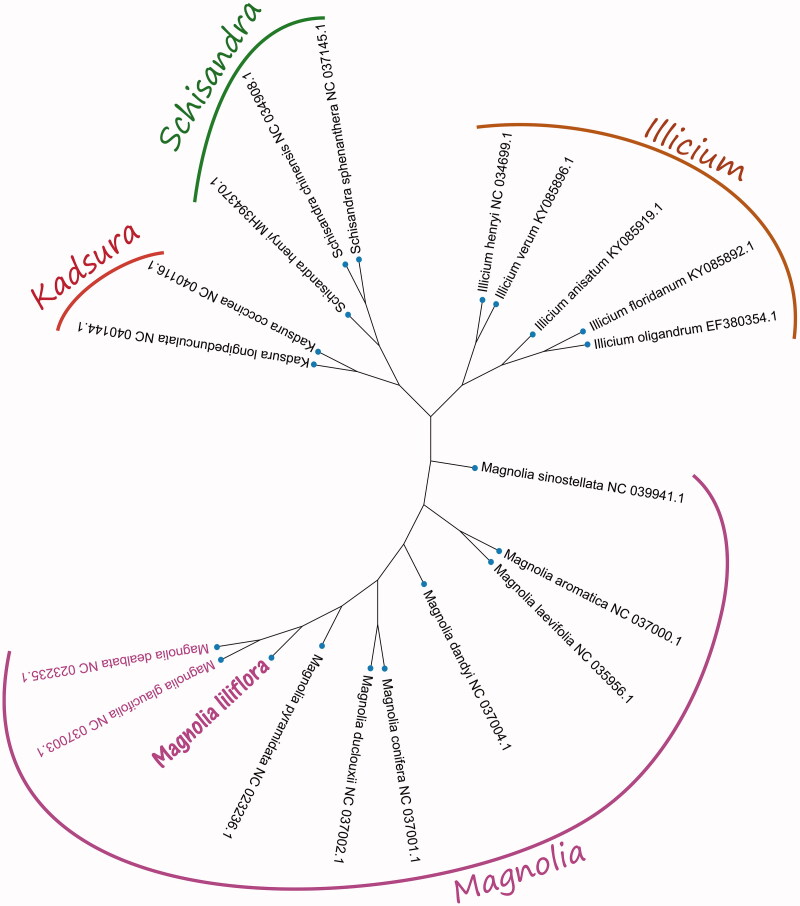
The maximum likelihood tree based on 20 species plants cp genomes. Shown next to the nodes are bootstrap support values based on 5000 replicates.
